# Criteria for indication and treatment modification in a cohort of patients with prostate cancer treated with hormone therapy

**DOI:** 10.1177/1756287218808496

**Published:** 2018-10-24

**Authors:** Thierry Lebret, Alain Ruffion, Igor Latorzeff, Marc Zerbib, Jean-Luc Moreau, Dominique Rossi, Nathalie Pello-Leprince-Ringuet, Valérie Perrot, Christophe Hennequin

**Affiliations:** Hôpital Foch, Université Versailles St. Quentin en Yvelines, Chef de Service Urologie et Transplantation Rénale, 40 Rue Worth, 92151 Suresnes, France; Centre Hospitalier Lyon Sud, Pierre Bénite, France; Polyclinique du Parc, Toulouse, France; Hôpital Cochin, Paris, France; Polyclinique Gentilly, Nancy, France; Hôpital Nord, Marseille, France; Ipsen Pharma, Boulogne-Billancourt, France; Ipsen Pharma, Boulogne-Billancourt, France; Hôpital Saint-Louis, Paris, France

**Keywords:** advanced prostate cancer, androgen deprivation therapy, gonadotropin-releasing hormone agonist, hormonal manipulation, treatment modification

## Abstract

**Background:**

No published studies have specifically assessed whether treatment modifications to androgen deprivation therapy (ADT) for prostate cancer (PCa) are frequently carried out in routine clinical practice. The current study was conducted to determine what proportion of patients who had initiated hormone therapy with a gonadotropin-releasing hormone (GnRH) analogue then had their treatment regimen modified during the first 24 months.

**Methods:**

A prospective, noninterventional study was carried out in routine clinical practice in France. Patients with locally advanced or metastatic PCa were followed up for 2 years after treatment initiation with a GnRH analogue. The primary endpoint was the proportion of patients with a modification to their initial hormone therapy.

**Results:**

In total, 1301 patients were enrolled into the study by 204 physicians, and the primary endpoint could be evaluated for 891 patients. The GnRH analogue treatment was initiated for metastatic PCa (24.2%), locally advanced PCa without planned local treatment (20.6%), locally advanced PCa in association with radiotherapy (31.6%), and biochemical recurrence after local treatment (21.4%). Hormonal treatment was modified in 43.8% (390/891) of patients during the 24-month follow-up period after GnRH analogue initiation. In 61.3% of cases (239/390), the type of modification involved a change of GnRH analogue formulation or switch to another GnRH analogue. A total of five significant predictive factors for GnRH analogue treatment modification were identified: metastatic stage; physician sector; physician speciality; presence or absence of urinary symptoms; and intermittent *versus* continuous ADT.

**Conclusions:**

This study shows that in 43.8% of the patients with advanced PCa, ADT is modified in the first 2 years after initiation in routine clinical practice. Predictive factors for alteration of ADT were metastatic stage and the choice of an intermittent schedule.

## Introduction

In France, as in many developed countries, prostate cancer (PCa) is the most frequent form of cancer in men, with an average age at diagnosis of 70 years.^1–4^ PCa is believed to have been responsible for 8900 deaths in France in 2012.^5^

Depending on the stage of the cancer, androgen deprivation therapy (ADT) can be prescribed in association with curative treatment or as a single therapy. In most cases, ADT consists of treatment with a gonadotropin-releasing hormone (GnRH) analogue, such as triptorelin, leuprorelin or goserelin. Treatment with a GnRH analogue can normalize serum prostate-specific antigen (PSA) levels in over 90% of patients,^6^ and disease regression will often occur. However, primary hormone sensitivity reduces over time, and most tumours will ultimately stop responding to treatment. Although the duration of response to ADT is highly variable, the average duration of response in metastatic disease is typically 18 months to 2 years.^7,8^ After the failure of primary ADT, secondary treatment options are recommended such as the addition to ADT of newer hormonal agents, abiraterone or enzalutamide, or chemotherapy.^9,10^

Intermittent ADT has been proposed to potentially improve patients’ quality of life by reducing adverse events (AEs) associated with androgen deprivation.^11–16^ However, recent evidence suggests that in some populations intermittent and continuous ADT are associated with similar long-term AEs, and intermittent ADT could even have a detrimental effect in metastatic disease.^17^

To our knowledge, no published studies have specifically assessed whether hormonal treatment alterations are frequently carried out in routine clinical practice, what such alterations entail, and the timing and reasons of such manipulations. The current study was conducted to determine, in routine clinical practice in France, what proportion of patients who had initiated hormone therapy with GnRH analogues for locally advanced or metastatic PCa then had their treatment regimen modified during the first 24 months. Reasons for these changes, predictive factors for treatment modification, and secondary treatment practices were also recorded.

## Methods

The study was noninterventional and adhered to all local regulatory requirements applicable to non-interventional studies [Comité Consultatif sur le Traitement de l’Information en Matière de Recherche dans le Domaine de la Santé (CCTIRS; French Advisory Committee on Data processing in Research in the Field of Health), the Commission Nationale de l’Informatique et des Libertés (CNIL; French Data Protection Authority) and the Conseil National de l’Ordre des Médecins (CNOM; National Board of the French Medical Association)]. The study was conducted according to the ethics and Good Epidemiological Practice recommendations developed by the Association of French-Language Epidemiologists (ADELF). All patients gave written informed consent for their medical records to be accessed.

### Study design and data collection

A national, multicentre, noninterventional, longitudinal epidemiological study was conducted in France, in a representative population of men treated with a GnRH analogue for locally advanced or metastatic PCa, conducted under the aegis of the Association Française d’Urologie. The study ran from September 2011 to January 2015. Assessments were made at consultations scheduled by the participating physicians as part of the standard follow up for patients (usually every 6 months). All prescribed medications and medication changes were at the sole discretion of the physician and the patient.

Data available in the patients’ medical records and data collected for 24 months following inclusion as part of the routine management of their disease were used in the analysis. Data were collected on a patient case report form (CRF) at study inclusion and at the regular follow up visits. The CRF captured information pertaining to a change in hormonal therapy, as well as biochemical, clinical and laboratory variables. Nonserious and serious drug-related AEs [adverse drug reactions (ADRs)] were reported to the safety department of the drug manufacturer, using the usual process for reporting such events.

### Physician recruitment

It was expected that 300 physicians would be required to obtain a representative sample of men undergoing hormone treatment for PCa. The process of physician selection was conducted independently of the study sponsor by a contract research organization, ITEC Services (Cenon, France). Physicians were recruited from a regularly updated list (including 1709 specialists), developed by Cegedim (Boulogne-Billancourt, France) for Ipsen Pharma, of physicians who regularly prescribe GnRH analogues for locally advanced or metastatic PCa. An initial information letter describing the study was sent to physicians on the national list (by the scientific committee), and the first 300 physicians expressing an interest were selected to participate in the study.

### Selection of patients

Each participating physician was requested to recruit four consecutive patients who met the criteria for study entry. Inclusion criteria were: adult patients (⩾18 years of age); locally advanced or metastatic PCa; decision by the healthcare team that the patient requires hormone therapy; initiation of treatment with a GnRH analogue at inclusion in the study; and written informed consent to participate. Exclusion criteria included: treatment with a GnRH antagonist; treatment with anti-androgen monotherapy; participation in a clinical trial at the time of inclusion; or previous treatment with a GnRH analogue at the locally advanced or metastatic stage of disease within the previous 2 years.

### Statistical analysis

The primary endpoint was the proportion of patients with a modification to their initial hormone therapy (i.e. scheduled or unscheduled discontinuation of the treatment). Withdrawal of anti-androgen initially prescribed for a few weeks to prevent a flare-up was not considered as a hormonal modification. Secondary endpoints were: baseline patient and disease characteristics; details of GnRH analogue treatment at initiation (e.g. injection frequency); reasons for treatment changes; subsequent treatment strategies; predictive factors for treatment modification; progression of clinical or laboratory variables; and tolerability.

The three study populations were: the population of active physicians (all specialists who included at least one patient in the study), the study population (all patients enrolled in the study) and the modified study population (all patients who completed the follow up at 24 months, or had a modification to their initial hormone therapy before prematurely ending follow up, or died during the 24-month follow-up period).

In the absence of published information, a rate of treatment modification of 50% was assumed; from this, it was estimated that data from 1067 patients would be needed to give an absolute accuracy of 3%. Assuming approximately 15% of medical records would be unusable, it was calculated that a sample size of 1200 patients would be needed. The primary endpoint was described for the modified study population as the median and 95% confidence interval (CI) and was calculated using the Agresti–Coull method. Univariate analyses tested factors predicting hormone therapy treatment modification: physician characteristics, patients’ medical characteristics at baseline, cancer history and specifics, and hormonal treatment characteristics. After the univariate analysis a selection of variables was made for inclusion in the multivariate model. For the initial selection of variables, the level of significance was 20%. The stepwise selection of variables for the multivariate analysis was made at a significance level of 5%. Statistical analyses were performed using SAS^®^ software, version 9.1 or later (SAS Institute, NC, USA).

Since percentages were calculated from the answers provided on the CRF (excluding missing data), the figures do not always add up to 100%. Multiple responses to a single element of the CRF have produced percentages over 100%.

## Results

A total of 1301 patients with PCa were enrolled in the study by 204 French physicians. Most participating physicians were urologists (91.2%) and radiation oncologists (8.4%). Most physicians worked in private centres (60.3%); 33.8% worked in the mixed sector and 5.9% worked in a hospital. Most (77.9%) enrolled 4 or more patients, with a maximum number of 20 patients enrolled by any one physician.

### Patients

**Figure 1.**
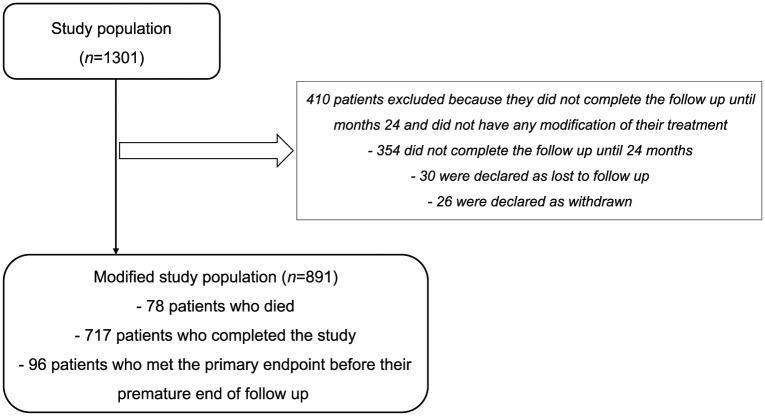
Participant flow chart.

**Figure 2.**
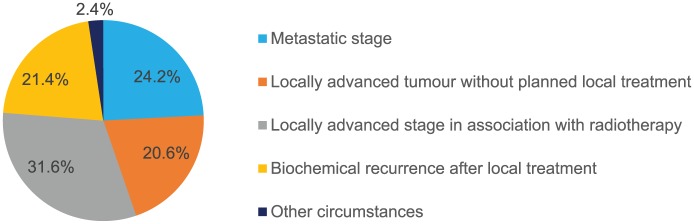
Circumstances of initiation of GnRH analogue therapy in the modified study population (*n* = 891). GnRH, gonadotropin-releasing hormone.

Of 1301 enrolled patients, 717 (55.1%) completed the study, 421 (32.4%) did not have a completed CRF, 78 (6.0%) died, 48 (3.7%) were lost to follow up and 37 (2.8%) withdrew. The modified study population consisted of 891 patients, comprising the 717 patients who completed the study, 78 patients who died during their follow up, and 96 patients who met the primary endpoint before their premature end of follow up (including 67 from those patients who did not have a completed CRF, 18 from those who were lost to follow up, and 11 from those who withdrew; Figure 1). Patient characteristics of the modified study population at baseline are listed in Table 1. The characteristics were similar between patients from the study population and patients from the modified study population. The circumstances of GnRH analogue therapy initiation are shown in Figure 2.

**Table 1.** Baseline patient and disease characteristics in the modified study population (*n* = 891).

Characteristic	Patients, *n* (%)*
Mean ± SD age at initial diagnosis of PCa, years	72.5 ± 8.9
Mean ± SD age at time of GnRHa treatment initiation, years	74.1 ± 8.7
Circumstances of PCa diagnosis	
Individual screening	527 (59.1)
Urinary disorders	180 (20.2)
Incidental finding after prostate resection	75 (8.4)
Bone metastases	55 (6.2)
Performance status impairment	57 (6.4)
Other circumstances^a^	72 (8.1)
Mean ± SD BMI, kg/m^2^	26.5 ± 3.6
WHO performance status	
0	401 (50.9)
1	261 (33.1)
2	80 (10.2)
3	31 (3.9)
4	15 (1.9)
At least one comorbidity or associated factor	645 (72.4)
Arterial hypertension	433 (48.6)
Dyslipidaemia	193 (21.7)
Diabetes	172 (19.3)
Ischaemic cardiomyopathy	161 (18.1)
Osteoporosis	11 (1.2)
Neuropsychological disorders	24 (2.7)
Treatment with long-term corticosteroids	7 (0.8)
Other clinically significant history	152 (17.1)
At least one urinary symptom	474 (53.2)
Pollakiuria	289 (32.4)
Dysuria	245 (27.5)
Nocturia	155 (17.4)
Urgent urination (urge incontinence)	114 (12.8)
Haematuria	29 (3.3)
Urinary incontinence	44 (4.9)
Urinary retention	51 (5.7)
At least one other clinical symptom	411 (46.1 %)
Erectile dysfunction	239 (26.8)
Asthenia	188 (21.1)
Bone pain	85 (9.5)
Anorexia	32 (3.6)
Other clinically significant symptoms	17 (1.9)
Sexually active	225 (26.0)
Median PSA level at inclusion, ng/ml [Q1; Q3]	17.0 [6.89; 47.39]
Gleason score at the time of diagnosis	
<7	151 (17.3)
7 (3+4)	227 (26.0)
7 (4+3)	202 (23.1)
>7	293 (33.6)
Missing	18
TNM stages of PCa at inclusion	
T0	12 (1.4)
T1–T2 N0M0	69 (7.8)
T3–T4 N0–xM0	396 (44.9)
All T, N1, M0	65 (7.4)
All T, All N, M1**	188 (21.3)
Other	151 (17.1)
Missing	10

* Unless otherwise stated in the left-hand column; total percentages may not equal 100% because of rounded figures or because of multiple answers possible for some parameters. Percentages are calculated on nonmissing data.

** 194 patients in M1 stage and 188 patients with M1 stage and both T and N stages filled.

a Includes recurrence after local treatment.

BMI, body mass index; GnRHa, gonadotropin-releasing hormone analogue; PCa, prostate cancer; PSA, prostate-specific antigen; SD, standard deviation; TNM, tumour, nodes, metastases; WHO, World Health Organization.

### GnRH analogue treatment initiation

The GnRH analogue prescribed at treatment initiation was triptorelin, leuprorelin or goserelin. Most patients started on a 3-month formulation (58.9%) or a 6-month formulation (40.2%), and the main route of administration was intramuscular (72.5%). Anti-androgens were administered to prevent or control flare-up in 52.6% of patients and were used to achieve complete androgen blockade (CAB) in 16.9% of patients. In most patients (87.6%), GnRH analogue treatments were administered continuously at initiation.

The decision to initiate therapy with a GnRH analogue was made during a formalized multidisciplinary team (MDT) meeting for most patients in the overall population (85.6%) and in the patient subgroup with treatment modification (84.7%). Subsequent treatment decisions were performed in MDT meetings for only 41.4% of the subgroup of patients who required treatment modifications. There were no relevant differences in treatment decisions according to whether the physician was based in a private centre, a mixed centre, or a public hospital.

### Modification of GnRH analogue treatment

In terms of the primary endpoint, hormonal treatment was modified in 43.8% (390/891) of patients during the 24-month follow-up period after GnRH analogue initiation. When subgroups were assessed according to the reasons for initiating GnRH analogue treatment, the proportion of patients with hormonal treatment modification ranged from 37.6% for patients who began GnRH therapy due to locally advanced disease in association with radiotherapy, to 49.7% in patients with biochemical recurrence after local treatment (Table 2).

**Table 2.** Modification of initial hormone therapy by reason for initiation of the current treatment with a GnRH analogue in the modified study population (*n* = 891).

Reason for initiation of GnRH analogue therapy	Initial hormone therapy modified *n* (%) [95% CI]
Missing (*n* = 7)	4 (57.1) [25.0; 84.3]
Locally advanced stage in association with radiotherapy (*n* = 279)	105 (37.6) [32.2; 43.5]
Locally advanced tumour without planned local treatment (*n* = 182)	70 (38.5) [31.7; 45.7]
Biochemical recurrence after local treatment (*n* = 189)	94 (49.7) [42.7; 56.8]
Metastatic stage (*n* = 214)	104 (48.6) [42.0; 55.3]
Other circumstances (*n* = 20)	13 (65.0) [43.2; 82.0]
Total (*n* = 891)	390 (43.8) [40.6; 47.1]

95% two-sided binomial confidence interval using Agresti–Coull method.

CI, confidence interval; GnRH, gonadotropin-releasing hormone.

Following univariate analysis and subsequent multivariate assessment, five significant predictive factors for GnRH analogue treatment modification were identified. Treatment was more likely to be modified if: the patient had metastatic PCa *versus* nonmetastatic (or unknown metastatic) status [48.6% *versus* 42.1%, respectively; odds ratio (OR) (95% CI): 1.539 (1.100; 2.155), *p* = 0.0119]; if the physician worked in a private centre compared with if they worked in the mixed sector [48.1% *versus* 36.1%, respectively; OR (95% CI): 1.553 (1.145; 2.105), *p* = 0.0005]; if the physician was a nonurologist compared with a urologist [58.1% *versus* 41.9%, respectively; OR (95% CI): 1.832 (1.145; 2.930), *p* = 0.0115]; if there were no urinary symptoms compared with at least one urinary symptom [51.1% *versus* 37.3%, respectively; OR (95% CI): 1.698 (1.277; 2.257), *p* = 0.0003]; and if the patient was prescribed ADT intended to be intermittent compared with those receiving continuous therapy [53.3% *versus* 42.6%, respectively; OR (95% CI): 1.598 (1.016; 2.418), *p* = 0.0420]. Of the 390 patients who had their treatment modified during the 24 months of the study, a change of the GnRH analogue formulation or a switch to another GnRH analogue (*n* = 239) was the most frequent form of treatment modification, regardless of the reason for initiation of GnRH analogue therapy (Table 3). No statistical comparison of subgroups was done. The main difference (based on descriptive data) was on chemotherapy, which was initiated at the metastatic stage in all except two patients. The exact reason for unscheduled modification of initial hormone therapy was recorded in 110 patients: the main reasons were progression (55.5%), mainly biochemical progression with or without clinical or radiological progression (50.9%), and patient’s decision (26.4%) as shown in Table 4.

**Table 3.** Type of treatment modification among those patients whose ADT was modified in the 24 months after treatment initiation: for all patients (*n* = 390) and in subgroups according to reason for initiation of GnRH analogue therapy.

Type of treatment modification *n* (%)	Locally advanced stage in association with radiotherapy (*N* = 105)	Locally advanced tumour without planned local treatment (*N* = 70)	Biochemical recurrence (*N* = 94)	Metastatic stage (*N* = 104)	All** (*N* = 390)
Change of GnRH analogue formulation or switch to another GnRH analogue	68 (64.8)	47 (67.1)	55 (58.5)	57 (54.8)	239 (61.3)
– Change of GnRH analogue formulation	30 (28.6)	25 (35.7)	34 (36.2)	27 (26.0)	122 (31.3)
– Switch to another GnRH analogue	38 (36.2)	22 (31.4)	21 (22.3)	30 (28.8)	117 (30.0)
Change of the planned duration of GnRH analogue treatment	9 (8.6)	6 (8.6)	16 (17.0)	8 (7.7)	41 (10.5)
Change to intermittent treatment	6 (5.7)	13 (18.6)	16 (17.0)	4 (3.8)	39 (10.0)
Initiation of chemotherapy	2 (1.9)	0	0	20 (19.2)	22 (5.6)
Hormonal manipulation	4 (3.8)	11 (15.7)	6 (6.4)	14 (13.5)	35 (9.0)
– Addition of an anti-androgen	2 (1.9)	9 (12.9)	6 (6.4)	4 (3.8)	21 (5.4)
– Withdrawal of anti-androgen as part of CAB*	2 (1.9)	2 (2.9)	0	6 (5.8)	10 (2.6)
– Addition of oestrogen therapy	1 (1.0)	0	0	9 (8.7)	10 (2.6)
Inclusion in another clinical trial	0	0	0	1 (1.0)	1 (0.3)
Other treatment strategy***	20 (19.0)	7 (10.0)	13 (13.8)	18 (17.3)	63 (16.2)

* Only withdrawal of anti-androgen as part of CAB was considered as treatment modification; withdrawal of anti-androgen prescribed initially for a few weeks to prevent flare-up was not considered as treatment modification.

** For 17 patients the reason for initiation of GnRH analogue therapy was ‘other’, they are therefore not included in the subgroups columns.

*** Most other treatment strategies were unspecified, 23 out of 63 were prescriptions of new generation hormonal treatment or radiotherapy.

Multiple answers were possible for some parameters, hence the total percentages may not equal 100%.

ADT, androgen deprivation therapy; CAB, complete androgen blockade; GnRH, gonadotropin-releasing hormone.

**Table 4.** Reasons for unscheduled modification of initial hormone therapy among the subgroup of patients with specification of a reason for unscheduled modification (*n* = 110).

Parameter	Patients *n* (%)
Progression	61 (55.5)
Biochemical progression	56 (50.9)
Clinical progression	27 (24.5)
Radiological progression	27 (24.5)
Patient’s decision	29 (26.4)
Adverse events related to the treatment	14 (12.7)
Failure	8 (7.3)
Other reason	12 (10.9)

Multiple answers possible for some parameters, hence total percentages may not equal 100%.

### Biochemical, clinical and laboratory variables

In the modified study population, median PSA levels declined upon initiation of GnRH analogue therapy and remained low over the 24-month follow-up period (Table 5). The proportion of patients with urinary symptoms decreased from 53.2% at inclusion to 34.6% at 24 months, whereas the proportion of patients with other clinical symptoms increased from 46.1% to 49.0% during GnRH analogue treatment. The proportion of patients with erectile dysfunction increased from 26.8% to 35.1% during the study. Bone densitometry was performed in only 10 patients at baseline and between six and 20 patients over the 24-month follow-up period. It is therefore not possible to draw any conclusions on the possible effect of GnRH therapy on bone mineral density. There were no clinically relevant changes in other laboratory variables (cholesterol, liver enzymes, triglycerides, and fasting glycaemia) during the study.

**Table 5.** Median serum PSA levels from baseline to 24 months in the modified study population (*n* = 891).

	Inclusion (*n* = 891)	6 months (*n* = 869)	12 months (*n* = 863)	18 months (*n* = 874)	24 months (*n* = 875)
Median PSA ng/ml [Q1; Q3]	17.00 [6.89; 47.39]	0.24 [0.03; 1.70]	0.16 [0.03; 1.24]	0.14 [0.03; 1.20]	0.10 [0.03; 1.20]

PSA, prostate-specific antigen.

### Tolerability

AE reporting followed regulations relating to spontaneous reports; thus, if a significant new safety event had occurred, it would have been captured, processed and reported to the regulatory agencies in the usual manner. No safety issues arose from this study that required further investigation. All ADRs that occurred during this study were known side effects of androgen deprivation and have been described as very common (>10% of patients: hot flushes, erectile dysfunction) or common (<10% of patients: asthenia).

## Discussion

In this large observational study, 1301 patients with PCa were enrolled by more than 200 French physicians (mostly urologists). In the modified study population of 891 patients, baseline patient characteristics, such as an average age of 74.1 years, were generally representative of earlier reports of the typical French PCa patient population.^1,2,18,19^ Circumstances of PCa diagnosis in this population aligned with European Association of Urology (EAU) guidelines, which support PSA testing in men at elevated risk (risk factors include age >50 years).^10^ At initial diagnosis of PCa (i.e. before the baseline of this study, which was the time of GnRH analogue therapy initiation), patients from this observational study had an average age of 72.5 years and most had been identified because of individual screening.

The four main indications for initiating hormonal therapy occurred in similar proportions (Figure 2): metastatic PCa (24.2%), locally advanced PCa without planned local treatment (20.6%), locally advanced PCa in association with radiotherapy (31.6%), and biochemical recurrence after local treatment (21.4%). Another recent observational study in France assessing reasons for initiating GnRH analogue treatment found that 23.2% of patients had metastatic PCa, 19.9% had recurrence after local treatment and 44.2% had locally advanced PCa and were receiving local treatment.^19^ Another French observational study did not classify patients in the same way but 24.9% of patients initiating GnRH analogue treatment had metastatic PCa.^20^ These proportions are broadly consistent with those of the current study. Similarly, an international observational study that included information on the reasons for starting treatment with the GnRH analogue triptorelin, recorded metastatic PCa as the primary reason in 19.9% of patients and locally advanced PCa in 55.8%.^21^ However, in our study, the high rate of patients receiving a hormonal treatment for a locally advanced disease without a planned local treatment is a concern. It has been demonstrated that hormonal treatment alone is inferior to a combination of ADT and radiotherapy.^22,23^ It is not clear, in the current study, if the local treatment was not planned at the time GnRH analogue treatment initiation but would be subsequently organized, or if it was not planned at all. Cross-study comparisons of the indications for initiating GnRH analogue therapy are limited by differences in inclusion criteria and methods of collecting data, but it appears that there is a consistency between studies in France collecting data on everyday practice between 2009 and 2014. The possible impact of the STAMPEDE^24^ and CHAARTED^25^ study results would not be captured in these studies.

Overall, 43.8% of patients starting GnRH analogue treatment for locally advanced or metastatic PCa had a modification of their initial treatment during the first 24 months of treatment. A recent observational study in France reported 18.8% of patients having their GnRH analogue treatment regimen modified in the first 12 months.^19^ As above, the different methods employed in these studies make comparisons difficult, but it would not be surprising if the ADT was modified more in the second year than the first year after initiation. Treatment modifications in the current study mainly pertained to a change or switch in hormone therapy formulation, this means that most of the patients either were maintained under their initial GnRH analogue treatment (56.2%) or were changed of GnRH analogue but maintained under ADT (26.8%).

It should be noted that the reason for treatment modification was documented in only 110 of the 390 patients with treatment modification (Table 4). When documented, the main reason was biochemical progression (50.9%). Treatment modifications also occurred due to AEs related to therapy (12.7%) and to the patient’s decision (26.4%). It may be common practice in some centres to change the route of administration or type of GnRH analogue for these reasons with the aim of optimizing long-term adherence, efficacy and tolerability.

Treatment modifications occurred at numerically higher rates in patients receiving GnRH analogue therapy for metastatic disease or after biochemical recurrence than in those with locally advanced disease, either in association with radiotherapy or when no local treatment was planned (Table 2). This is perhaps not surprising and may indicate that management of more advanced disease is more problematic, with more ADT manipulations being needed in the first 2 years to control the disease. Other predictors of treatment modification were treatment at a private centre, treatment by a nonurologist, absence of urinary symptoms, and use of an intermittent ADT regimen. A higher likelihood of treatment modifications at a private centre compared with other settings may be due to economic factors, as financial considerations will differ between private and public healthcare settings.

Hormonal manipulation (addition or withdrawal of anti-androgen treatment or addition of oestrogen) occurred in 9.0% of patients. This low figure is unsurprising given that guidelines now recommend the addition of newer agents when progression occurs. One would expect that this figure would have been higher in the period before the introduction of abiraterone and enzalutamide, when secondary hormonal approaches were the standard of care for localized castrate resistant PCa (CRPC).^26^ The availability of newer agents for PCa such as abiraterone and enzalutamide may greatly alter the willingness of physicians to change ADT regimens, and will certainly alter subsequent treatment regimens. Indeed, guidelines now recommend the use of these life-prolonging agents upon the development of CRPC, and to maintain a backbone of ADT in this setting.^8–10^ This study spanned the period before and after the introduction of abiraterone and enzalutamide; however, no attempt was made to assess the rate of hormonal manipulation early in the data collection period compared with later in the study.

Initiation of chemotherapy was the reason for hormonal modification in just 5.6% of patients, probably because chemotherapy is mostly prescribed as an add-on treatment to ADT. A recent meta-analysis pooled the results from three trials (CHAARTED, GETUG-15 and STAMPEDE) and showed that in hormone-naïve metastatic PCa, the addition of chemotherapy (docetaxel) to ADT conveyed an overall survival benefit (hazard ratio, 0.77; *p* < 0.0001). This translated to a 10% absolute improvement in survival at 4 years. The authors concluded that adding docetaxel to a backbone of ADT should become the new standard of care for men with metastatic PCa who are fit enough to receive chemotherapy.^27^ Future studies will help to assess the impact of newer data such as this on routine clinical practice, and the observational study described here may serve as a ‘baseline’ to help assess such changes.

GnRH analogues were generally administered continuously (in 87.6% of patients) and intramuscularly (72.5%). A total of 69.5% of patients also received an anti-androgen, mostly to reduce flare during the first weeks of treatment. This is in line with a previous study in which 66.7% of patients were prescribed an anti-androgen at initiation of GnRH analogue therapy.^19^ In the current study, the 3-month GnRH analogue formulations were used slightly more frequently than 6-month formulations (58.9% *versus* 40.2%, respectively), whereas another recent French observational study indicated more frequent treatment initiation with a 6-month formulation (62.8% *versus* 37.2% with a 3-month formulation).^19^

Urinary symptomology was reduced after the initiation of GnRH analogue treatment (53.2% of patients had at least one urinary symptom at inclusion *versus* 34.6% at 24 months), reflecting a general decrease in all urinary symptoms. This is consistent with a previous study in which triptorelin was shown to be effective in improving lower urinary tract symptoms in men with locally advanced or metastatic PCa, as demonstrated by a reduction in the International Prostate Symptom Score.^21^

Limitations that are common to observational studies were present in this study. For example, since all data were collected in accordance with daily clinical practice, if specific assessments were not routinely performed by the physicians, this led to missing data on the CRF. A further limitation was the small number of patients with a reason for modification of their hormonal treatment (*n* = 110); this means that caution must be taken when extrapolating these findings to the wider population. Likewise, data on the timing of treatment modification was available in a small minority of patients, and so we have not reported this information. Nevertheless, each CRF with a modification was individually re-read to confirm the type of modification and the reason; thus, despite the observational nature of the study, care was taken to carefully check the results. Furthermore, quality control was carried out at 10% of the sites as standard. Despite its weaknesses, this observational study provides useful findings on the frequency of hormonal treatment modification in the 24 months after initiating GnRH analogue therapy in patients with locally advanced or metastatic PCa.

National and international recommendations specify that a modification of hormonal treatment could be proposed when there is resistance to castration, inefficiency of the GnRH analogue to obtain a testosterone level <0.5 ng/ml or poor tolerability.^9,10^ In this observational study, most of the modifications were in accordance with these recommendations,^9,10^ with patient decisions also making an important contribution.

## Conclusion

In this large French observational study, 43.8% of patients with advanced PCa had modification to their ADT during the 24 months of follow up. Modifications mainly consisted of a simple change in the formulation or type of GnRH analogue, with the patient otherwise being maintained on ADT. Predictive factors for alteration of the ADT included metastatic stage and the choice of an intermittent schedule. As most of the patients were maintained under their initial GnRH analogue treatment (58.2%) or were changed of GnRH analogue but maintained under ADT (26.8%), this study confirms that ADT remains the backbone therapy of advanced PCa in routine clinical practice.
